# A Review of Genetic and Physiological Disease Mechanisms Associated With Cav1 Channels: Implications for Incomplete Congenital Stationary Night Blindness Treatment

**DOI:** 10.3389/fgene.2021.637780

**Published:** 2021-01-28

**Authors:** Tal T. Sadeh, Graeme C. Black, Forbes Manson

**Affiliations:** ^1^Division of Evolution and Genomic Sciences, Faculty of Biology, Medicine and Health, University of Manchester, Manchester, United Kingdom; ^2^Manchester Centre for Genomic Medicine, Manchester Academic Health Sciences Centre, Manchester University NHS Foundation Trust, St Mary’s Hospital, Manchester, United Kingdom

**Keywords:** L-type calcium channels, mutation analysis, incomplete congenital stationary night blindness, treatment, CaV1.4 calcium channel

## Abstract

Calcium channels are crucial to a number of cellular functions. The high voltage-gated calcium channel family comprise four heteromeric channels (Cav1.1-1.4) that function in a similar manner, but that have distinct expression profiles. Three of the pore-forming α_1_ subunits are located on autosomes and the forth on the X chromosome, which has consequences for the type of pathogenic mutation and the disease mechanism associated with each gene. Mutations in this family of channels are associated with malignant hyperthermia (Cav1.1), various QT syndromes (Cav1.2), deafness (Cav1.3), and incomplete congenital stationary night blindness (iCSNB; Cav1.4). In this study we performed a bioinformatic analysis on reported mutations in all four Cav α_1_ subunits and correlated these with variant frequency in the general population, phenotype and the effect on channel conductance to produce a comprehensive composite Cav1 mutation analysis. We describe regions of mutation clustering, identify conserved residues that are mutated in multiple family members and regions likely to cause a loss- or gain-of-function in Cav1.4. Our research highlights that therapeutic treatments for each of the Cav1 channels will have to consider channel-specific mechanisms, especially for the treatment of X-linked iCSNB.

## Introduction

Voltage-gated calcium channels perform multiple functions including signaling, hormone and neurotransmitter secretion, muscle contraction, and gene expression (Catterall et al., 2005). The family is grouped on the voltage sensitivities of the α_1_ subunits, with Cav1.1-1.4 comprising the high voltage L-type channels (Catterall et al., 2005). Cav1.1-1.3 channels are encoded by autosomal genes; the Cav1.4 channel is encoded by the X-linked *CACNA1F* gene. Cav1 channels diverged from a common evolutionary ancestor and have 60–75% overall polypeptide sequence identity.

Within the voltage-gated calcium channels, the α subunit forms the conductive pore. It associates with a β subunit, an α_2_δ subunit, and, for some channels, a γ unit, in an equal ratio (Catterall et al., 2005; Supplementary Figure 1). The α_1_ protein structure consists of four homologous transmembrane repeats (I–IV), each comprising of six helical segments (S1–S6) connected by extracellular or cytoplasmic loops. The fourth segment of each repeat carries positively charged amino acids at every third position that function as voltage-sensor domains (VSDs) sensitive to membrane depolarization impulses (Catterall et al., 2005). The S5 and S6 segments form the pore, which is lined by the S5–S6 loops. Each of these S5–S6 loops contains a conserved glutamate residue that forms a negatively charged ring that functions as the ion-selectivity filter (Yang et al., 1993). Thus, their conserved properties enable the four Cav1 channels to function in a similar fashion, albeit in different temporo-spatial circumstances, for calcium homeostasis (Zamponi et al., 2015): The α_1_ subunit of Cav1.1 is encoded by the *CACNA1S* gene located on chromosome 1q32.1 and is exclusively expressed in skeletal muscle (Catterall et al., 2005). The Cav1.4 α_1_ subunit is located at Xp11.23 (encoded by *CACNA1F*) and is expressed in retinal interneurons (Bech-Hansen et al., 1998). Cav1.2 (*CACNA1C*, Chr12p13.33) and Cav1.3 (*CACNA1D*, Chr3p21.1) are expressed in many electrically excitable cells and are often expressed in the same cell (e.g., adrenal chromaffin cells, sinoatrial node, neurons, and atrial cardiomyocytes) (Zamponi et al., 2015). The channels’ unique tissue expression profiles are reflected in their different voltage-dependencies i.e., they activate and inactivate at different voltages.

Missense mutations in the autosomal Cav1.1-1.3 α genes, although variously described as gain-of-function (GoF) and loss-of-function (LoF), mostly cause a gain of activity that is associated with autosomal dominant phenotypes. For instance, Timothy syndrome (TS) is an autosomal dominant, multiorgan condition, that is predominantly caused by GoF Cav1.2 mutations [e.g., p.Gly406Arg (Splawski et al., 2005)]. A smaller number of LoF variants have been described and are generally associated with autosomal recessive traits, such as Cav1.3 insertion mutations that result in autosomal recessive congenital deafness (Baig et al., 2011).

X-linked congenital stationary night blindness (X-linked iCSNB) is a static monogenic disorder that results in visual disability predominantly in males. Pathogenic *CACNA1F* mutations disrupt Cav1.4 function and impair normal retinal synaptic transmission (Strom et al., 1998). There is a small number of reports of affected females, presumably as a result of skewed X-inactivation (Rigaudière et al., 2003; Hemara-Wahanui et al., 2005). In the majority of cases, recessive X-linked mutations in *CACNA1F* abolish or decrease Cav1.4 calcium current density. Missense mutations can increase or decrease current density. Null (amorphic) alleles abolish calcium currents, and hypomorphs reduce currents by either dysregulating the current window (e.g., a shift in the voltage dependence of activation or inactivation) or the quantity of calcium influx. These changes may be through the production of no active protein products (null), or by reducing transcription or producing a protein lacking full functionality (hypomorph). By contrast, hypermorphs result in hyperactive channels by either increasing the current window or calcium influx. These consequences are seen in electrophysiological recordings by a shift in the voltage dependence of activation or inactivation (hyperpolarized leftward shift or depolarized rightward shift), or by changing the amount of calcium influx.

In this study, we collate and analyze reported mutations in the α_1_ subunit-encoding genes of L-type channels to identify the similarities and differences between the autosomal Cav1.1-1.3 and X-linked Cav1.4 channels with the aim to inform the pathophysiology of Cav1.4 variants, which is an important prerequisite for future therapeutic intervention.

## Materials and Methods

### Reported Mutations

Mutations in the α_1_ subunits of Cav1.1-1.4 genes were retrieved from the Human Gene Mutation Database (HGMD, licensed version accessed on June 2020; Stenson et al., 2017) and functional studies were collated from PubMed. These were classed as missense, nonsense, splicing, deletion, insertion/duplications, or other (complex rearrangements or regulatory substitutions). The associated phenotypes and mode of inheritance were recorded from HGMD and Online Mendelian Inheritance in Man (OMIM; Amberger et al., 2009).

### Population Database Search (gnomAD)

The tolerance and constraint scores of mutation types in Cav1 genes and their minor allele frequencies (MAFs) in the general population were derived from The Genome Aggregation Database (gnomAD; Karczewski et al., 2019). The most common version of a gene in a population is referred to as the wildtype allele and variations are annotated relative to it; the MAF is the number of times a variant allele occurs in a population for any data set. A high Z score indicates more constraints and intolerance to synonymous and missense variations, and pLI score close to 1 implies that the gene is intolerant to protein-truncating variants (i.e., nonsense, frameshift, splice sites variants), which likely cause LoF (predicted LoF; pLoF). In addition, the observed/expected (oe) ratio compares the observed pLoF to the expected frequency of the variation in the general population, supporting the Z and pLI probabilities (90% CI).

### Physicochemical and Pathogenicity Prediction

The physicochemical properties of each mutation were manually analyzed using NCBI’s Amino Acid Explorer tools “Structure and Chemistry” and “Common Substitutions” (Bulka et al., 2006).^1^ These tools compare specific physicochemical constraints of the amino acid pair such as a change in amino acid size, charge, and hydrophobicity. The latter tool relies on BLOSUM62 matrix to sort the frequency of the substitution (Henikoff and Henikoff, 1992). Pathogenicity was predicted using polymorphism phenotyping v2 (PolyPhen-2; Adzhubei et al., 2010), which considers protein structural properties such as amino acid surface area accessibility and generates a score where 1 is damaging and 0 is benign.

UniProt was used to annotate channel-specific domains and amino acids (The UniProt Consortium, 2018). Functionally characterized mutations were assigned as null, hypomorphic, or hypermorphic, dependent on the published electrophysiological properties and protein expression.

### Conservation Analysis

Evolutionary constraints were analyzed by comparing protein conservation between Cav1 paralogs (protein accession numbers: Cav1.1 NP_000060.2; Cav1.2 NP_955630.3; Cav1.3 NP_000711.1; and Cav1.4 NP_005174.2) and 10 orthologs (*Mus* musculus, *Rattus* norvegicus, *Canis* lupus familiaris, *Felis* catus, *Macaca* mulatta, *Sus* scrofa, *Danio* rerio, *Halichoerus* grypus, *Zootoca* vivipara, and *Xenopus* tropicalis), using Clustal Omega (Sievers et al., 2011). *Xenopus* was not included for Cav1.3 as only low-quality sequences are available.

## Results

### Cav1 Proteins: Incidence of Loss-of-Function (LoF) Mutations

For the four Cav1 encoding genes the population frequencies of LoF variants were examined and respective scores were ascertained from gnomAD. Cav1.1 has a total of 76 different LoF variants recorded on gnomAD with pLI = 0; oe = 0.39; the total number of Cav1.1 LoF alleles was 686. The low pLI score predicts that Cav1.1 is tolerant to LoF variants and therefore tolerant of haploinsufficiency (i.e., there will be sufficient protein function from the remaining wildtype allele). By contrast, Cav1.2 and Cav1.3 have a lower incidence of LoF variations in the population than Cav1.1. There are 31 LoF variants for Cav1.2, comprising 81 alleles (pLI = 1; oe = 0.1). For Cav1.3 there are 36 different LoF allele that collectively occur 296 times (pLI = 1; oe = 0.21). The low incidence of LoF variants in these channels indicates they are less tolerant of haploinsufficiency. It should be stressed that despite these statistical predictions only functional analyses will confirm if a LoF variant is associated with a loss of protein function, i.e., reduced current density and changes in protein expression. In Cav1.4 there are 35 different LoF variants collated in gnomAD, with an allele count of 54. Most of these occur only once in a single individual with 17 hemizygote males and 37 heterozygote females. A LoF pLI = 0 and oe = 0.45 indicates that Cav1.4 is not under selection against such variants.

### Cav1 Mutation Spectrum in Disease

**FIGURE 1:**
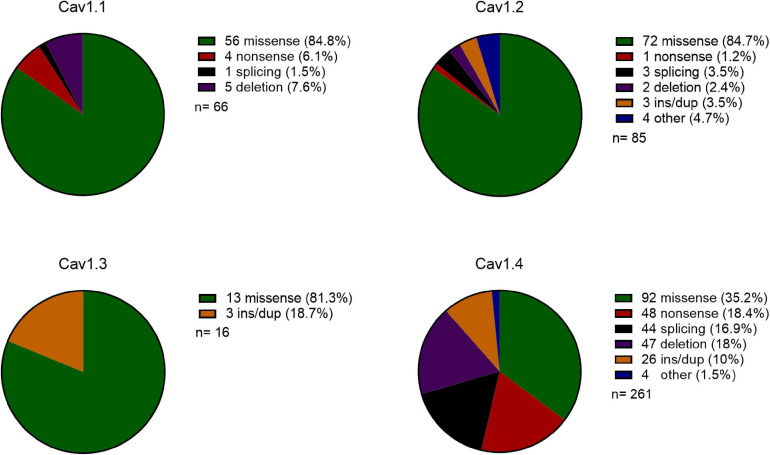
Comparison of the type of mutations in Cav1 genes reported on HGMD. Pie chart of the type of mutations cataloged for Cav1.1-1.4 genes. Mutation key and numbers are shown on the right: ins/dup: insertion/duplication, other: complex rearrangements or regulatory substitutions, n: number of reported mutations.

Overall, for monogenic disorders caused by pathogenic variation in Cav 1 channels, the proportion of missense vs LoF variants is very different (Figure 1). Cav1.1-1.3 have 0–6% nonsense mutations, whereas, Cav1.4 has 18% nonsense mutations. Cav1.1-1.3 have 81–85% missense mutations and Cav1.4 has 35% missense mutations.

#### Cav 1.1

There are 66 Cav1.1 mutations reported in HGMD, of which, 56 are missense mutations (85%). These missense alterations are the sole group of pathogenic variants associated with common Mendelian Cav1.1 conditions: malignant hyperthermia (MH; Monnier et al., 1997), muscular dystrophy (Zenagui et al., 2018), primary and hypokalemia periodic paralysis (hypoPP; Ptácek et al., 1994), exertional heat illness (Fiszer et al., 2015), and rhabdomyolysis (Vivante et al., 2017). These all follow an autosomal dominant inheritance, apart from muscular dystrophy and rhabdomyolysis, which are also inherited recessively. Overall, 60% of the reported mutations result in MH (16 mutations), myopathy (12 mutations), and hypoPP (11 mutations), with hypoPP being a monogenic condition; caused by Cav1.1 mutations only.

The 10 reported LoF variants (five deletions, four nonsense, one splicing) have been associated with complex and multifactorial diseases such as autism spectrum disorder (ASD) (Torrico et al., 2019), schizophrenia (Purcell et al., 2014), exertional heat illness, and myopathy (Hunter et al., 2015). While such Cav1.1 variants can be considered risk factors for these disorders, it is not possible to make definitive correlations due to variable expression and incomplete penetrance.

Six missense mutations in Cav1.1 have previously been functionally characterized *in vitro* – these are associated with hypoPP and MH (Supplementary Figure 2A). Interestingly, these result in both hypermorphic (one mutation) and hypomorphic (five mutations) proteins, although the latter are the most common (Table 1). Mutations reducing the current density are due to the loss of a positive charge in S4 VSDs, and three of these also delay the time course of activation [p.Arg528His (Morrill et al., 1998), p.Arg1239His and p.Arg1239Gly (Morrill and Cannon, 1999)].

**TABLE 1:** Summary of functionally analyzed Cav1 missense mutations.

Channel (gene)	Number of tested mutations	Effect on current	Details
Cav1.1 (*CACNA1S*)	6	5 decreased currents. 1 increased currents.	1 null and 4 hypomorphs are all Arg substitution that reduce the current density due to the loss of charge in the VSDs.
			1 hypermorph in S5-S6 loop lines the pore and results in faster activation of currents upon depolarisation.
Cav1.2 (*CACNA1C*)	17	5 decreased currents. 12 increased currents.	2 hypermorph in S6 affect the pore charge or hydrophobicity.
			14 mutations are in cytoplasmic loops: 10 hyper- and 4 hypo-morphs.
			4/5 hypomorphs affect trafficking and reduce membrane expression.
			All hypermorphs causing LQT8 increase the current window, which is consistent with a long QT interval.
			p.Ile1186Thr increase the current window and causes both LQT8 and TS but substitution to Val only seen in LQT8.
Cav1.3 (*CACNA1D*)	14	2 decreased currents. 12 increased currents.	6 hypermorph in S6 affect the pore charge or hydrophobicity and cause 3 phenotypes.
			2 hypomorph mutations: 1 Arg substitution reduces the current density due to the loss of charge in the VSDs. 1 His substitution unbalances the charge on an extracellular loop and reduces expression on the membrane.
			6 hypermorphs are in cytoplasmic loops that mostly cause a hyperpolarised shift.
Cav1.4 (*CACNA1F*)	10	7 decreased currents. 3 increased currents.	3 hypermorph in S6 affect the pore charge or hydrophobicity.
			3 hypomorphs mutations: 2 affect trafficking and reduce protein expression. 1 reduce the current density with normal protein expression.
			4 null mutations have no currents with normal protein expression. (p.Gly1007Arg introduces an extra charge to the VSDs).

*VSDs, voltage sensor domains, LQT8, Long QT syndrome 8, TS, Timothy syndrome. Additional details in Supplementary Table 1.*

Two of the functionally analyzed mutations (p.Arg175Trp, p.Arg1086His) are reported in the heterozygous state in gnomAD and have very low MAFs (0.00003 and 0.000004, respectively); no other analyzed mutation is present in gnomAD.

#### Cav1.2

There are 85 Cav1.2 mutations reported in HGMD, with 72 missense mutations (85%). These result in three monogenic cardiovascular disorders including Long QT syndrome 8 (LQT8; Boczek et al., 2013), Brugada syndrome 3 (BRGDA3; Antzelevitch et al., 2007), and TS (Splawski et al., 2004), which are autosomal dominant traits. These account for 60% of the reported mutations: LQT8 (25 mutations), BRGDA3 (12 mutations), and TS (11 mutations). In addition, Cav1.2 mutations cause a range of common disorders including cardiomyopathy (D’Argenio et al., 2014), atrial or ventricular fibrillation (Maltese et al., 2019), bradycardia (Zhu et al., 2018), and cerebellar ataxia (Chen et al., 2019), which are both autosomal dominant and recessively inherited. Cav1.2 has been reported in association with four complex diseases: ASD (Jiang et al., 2013), intellectual disability (Hu et al., 2019), schizophrenia (Roussos et al., 2014), and epileptic encephalopathies (Bozarth et al., 2018).

Missense mutations are the most frequent type associated with Cav1.2 disease. However, a small number of LoF variants (two splicing and one insertion mutations) are described in patients with BRGDA3 and cerebellar ataxia. Ten other mutation types have been described associated with two complex disorders; schizophrenia (including four regulatory substitutions, two deletions, one splicing, and one nonsense mutation), and ASD (two insertions). As for Cav1.1, the genetic heterogeneity of polygenic disorders makes it difficult to be conclusive regarding their correlation.

Seventeen Cav1.2 missense mutations have previously been functionally characterized by cell electrophysiology (Table 1). These are associated with BRGDA3, LQT8, and TS (Supplementary Figure 2B). Hypermorphs are the most common and mutations in transmembrane domains increase the current window by different mechanisms including a shift in the activation and/or inactivation or by increasing the maximum ion conductance (detailed in Supplementary Table 1). There are five hypomorphs that reduce the current density, most of which express poorly at the plasma membrane [p.Ala39Val (Antzelevitch et al., 2007), p.Asn300Asp (Béziau et al., 2014), p.Arg518Cys/His (Boczek et al., 2015)].

Three functionally analyzed mutations (two hypermorphs: p.Ala28Thr, p.Gly406Arg, and one hypomorph: p.Arg518His) are present in the general population with very low MAFs (0.00006, 0.0004, and 0.00003, respectively), and only in the heterozygous state. The other 13 functionally analyzed mutations are absent in gnomAD.

#### Cav1.3

Cav1.3 has 16 reported mutations in HGMD, of which, 13 are missense mutations (81%). These are associated with four Mendelian diseases including the autosomal dominant conditions primary aldosteronism with seizures and neurologic abnormalities (PASNA; Semenova et al., 2018), aldosterone-producing adenomas (APAs; Scholl et al., 2013), and epilepsy (Tumiene et al., 2018), and the autosomal recessive condition sinoatrial node dysfunction and deafness (SANDD; Liaqat et al., 2019). Five other complex conditions [ASD (O’Roak et al., 2012), bipolar disorder (Ross et al., 2016), developmental delays (Di Gregorio et al., 2017), and hearing impairment and intellectual disability (Garza-Lopez et al., 2018)] have been associated with Cav1.3 variants, but the exact causality remains unclear.

Missense mutations are the most frequent mutation type, however, three other mutation types have been associated with recessive (SANDD; one insertion) or complex (developmental delay; one insertion and ASD; one duplication) conditions.

The channel function of 14 missense mutations have previously been analyzed and are associated with APAs, ASD, hearing impairment and intellectual disability, and PASNA (Supplementary Figure 2C). Hypermorphs that increase the current density is the most frequent mechanism causing these conditions (Table 1). The hypomorphs have charge differences that reduce the current density.

None of the functionally analyzed mutations are present in gnomAD.

#### Cav1.4

Cav1.4 is the only X-linked Cav1 channel. 261 mutations have been reported in HGMD, of which, 92 are missense mutations (35%). 206 of all mutations are associated with iCSNB (Strom et al., 1998), the remainder causing cone-rod dystrophy (Jalkanen et al., 2006) and Aland island eye disease (Jalkanen et al., 2007). Retinitis pigmentosa (Xu et al., 2015), high myopia (Sun et al., 2015), and Usher syndrome (Song et al., 2011) have all been described although these remain unconfirmed.

About 169 truncating variants are predominantly associated with recessive iCSNB (48 nonsense, 42 deletions, 41 splicing, 25 insertion/duplications, and 4 complex rearrangements), and a small number are reportedly associated with cone-rod dystrophy [two deletions (Hauke et al., 2013; Huang et al., 2016) and one insertion (Jalkanen et al., 2006)] and Aland island eye disease [one deletion (Jalkanen et al., 2007)], or high myopia [one deletion and one splicing (Sun et al., 2015)] and retinitis pigmentosa (two splicing (Xu et al., 2015; Jespersgaard et al., 2019) and one deletion (Martin-Merida et al., 2019)].

Reduction or loss of activity from the single X chromosome allele is presumed to be the pathogenic mechanism for most truncating alleles in males since most will result in a lack of protein due to nonsense mediated decay (NMD; Sharma et al., 2020). The 48 nonsense alleles (out of 261 reported mutations, 18%) in Cav1.4 are predicted to be degraded through NMD. Nonsense mutations in the final exon often escape NMD, which may have different mechanistic consequences.

Ten iCSNB missense mutations have previously been functionally characterized. As expected for a gene associated with a large number of LoF mutations, hypomorphic alleles are the most common: seven hypomorphic or null alleles that reduce or abolish channel conductance. However, three hypermorphs increase channel conductance (Supplementary Figure 2D); these are in the S6 repeats. All mutations in loops are hypomorphic or null alleles (Table 1). The four null alleles express at normal global protein levels, which suggests that the proteins are unstable and escaped degradation and are not trafficked to the membrane correctly.

The null p.Gly1007Arg mutant has a very low MAF (0.00003) in the general population and there are no homozygous individuals. No other functionally analyzed mutations are present in gnomAD.

### Composite Cav1 Mutation Analysis

In order to understand the mechanistic consequences of missense alleles we next analyzed the missense mutations reported in the four Cav1 α_1_ genes. However, since causality is so hard in complex diseases we only looked at the mutations associated with verified monogenic phenotypes.

**FIGURE 2:**
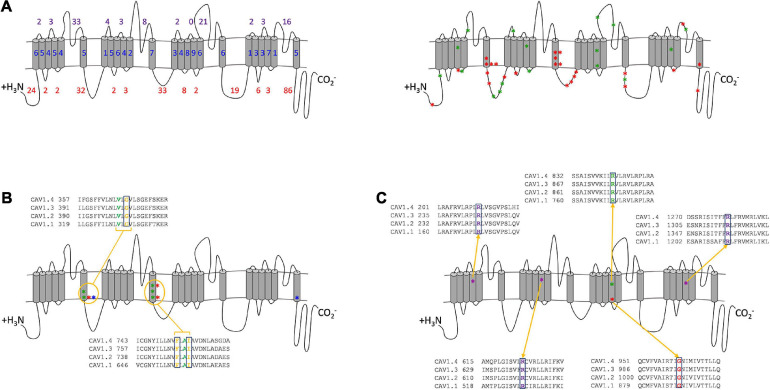
Combined analysis of Cav1 mutation positions. **(A)** Location of Cav1 α_1_ subunit mutations. **(Left)** The number of mutations in transmembrane (blue), extracellular (purple) and cytoplasmic (red) regions. **(Right)** The 47 functionally tested missense mutations are shown as red (GoF) and green (LoF) stars. Stars represent a mutated residue and may include multiple amino acid substitution. The cytoplasmic loops are the most frequently mutated regions (222 mutations), of which, 86 are in the C-terminal tail, whereas the extracellular loops have 97 mutations, and the transmembrane domains have 110 mutations. 114 mutations are in the pore-forming domains, and 25 are in the S4 VSDs. **(B)** Three functionally characterized hypermorphs in S6 repeats I and II of Cav1.4 [p.Gly369Asp (Hoda et al., 2005), p.Phe753Cys (Hope et al., 2005), p.Ile756Thr (Peloquin et al., 2007)] (red) are present at the equivalent position in Cav1.2: p.Gly402Ser (Splawski et al., 2005) (equivalent to p.Gly369Asp) (blue) and Cav1.3: p.Gly403Asp/Arg (Scholl et al., 2013) (equivalent to p.Gly369Asp), p.Ile750Met (Scholl et al., 2013) (equivalent to p.Ile756Thr), p.Phe747Leu (Pinggera et al., 2018) (equivalent to p.Phe753Cys) (green). **(C)** Five mutations to the VSDs (four hypomorphs and one null) are functionally analyzed mutants that damage the sensor’s voltage sensitivity and reduce the channel’s conductance in Cav1.1 [p.Arg174Trp (Bannister and Beam, 2013), p.Arg528His (Morrill et al., 1998), p.Arg1239His/Gly (Morrill and Cannon, 1999)] (purple), Cav1.3 [p.Arg990His (Monteleone et al., 2017)] (green), and Cav1.4 [p.Gly1007Arg (Peloquin et al., 2007)] (red). The conservation in paralogs is shown for each residue.

In total 429 mutations have been described in the Cav1 α_1_ genes and are associated with 35 phenotypes. 234 are missense variants, of which, 47 have previously been functionally characterized by *in vitro* assays and are pathogenic in 10 phenotypes (Figure 2). Of the functionally analyzed mutations, hyperactive channels are the most common and most are associated with autosomal dominant conditions, although this predominance is likely a result of bias by researchers selecting what mutants to study. Twenty-eight hypermorphic alleles increase the current density, and 19 hypomorphic or null alleles reduce it (Table 2). Supplementary Table 1 summarizes the functionally characterized mutations reported for Cav1 channels.

**TABLE 2:** Collated summary of Cav1 mutations.

Number of tested mutations	Effect on current	Details
47	19 decreased currents. 28 increased currents.	Cav1.1-1.3 are predominantly missense mutations (85%) and more tolerant of truncating mutations.
		Cav1.4 is intolerant to missense and truncating mutations.
		Cav1.1-1.3 mutations mostly cause hyperactive channels (n = 25 hyperactive and n = 12 hypoactive).
		Cav1.4 predominantly cause hypoactive channels (n = 7 hypoactive and n = 3 hyperactive).
		Mutations in S4 are hypomorphs – VSDs change of charge. n = 5 (3 in Cav1.1, 1 in Cav1.3, 1 in Cav1.4).
		Apart from S4, all mutations to helix are hypermorphs. n = 9 (2 in Cav1.2, 4 in Cav1.3, 3 in Cav1.4).
		Two hotspots in S6 of repeat I and II are all hypomorphs due to dysregulating the pore. n = 10 (1 in Cav1.2, 6 in Cav1.3, 3 in Cav1.4).
		Introducing a Pro constrains the loop resulting in less flexibility and hypomorphic alleles. Ser229Pro, Leu860Pro, Leu1068Pro (Cav1.4).
		Gly is important in a helix. Small size enables helix flexibility and substituting it results in hypermorphs. Gly402Ser (Cav1.2), Gly403Asp/Arg (Cav1.3), and Gly369Asp (Cav1.4).

*VSDs, voltage sensor domains. Detailed analysis on the effect on current is presented in Supplementary Table 1.*

By plotting all mutations on the composite Cav1 channel (Figure 2) it is possible to identify mutated residues that overlap between the channels and have specific consequences that are either shared or that are distinct between the channels. There are 46 missense mutations in the C-terminal tail, which may interfere with the calcium-dependent inactivation (CDI) machinery. The unique regulatory functions of this region in Cav1.4 suggest that mutations will have different effects in Cav1.1-1.3 than in Cav1.4.

There are 114 pathogenic mutations in the pore-forming domains (S5, S6, and S5–S6 loops), 10 of which are transmembrane and have been functionally analyzed *in vitro* (Figure 2B). Most of these amino acid substitutions result in a charge change (e.g., uncharged non-polar glycine to positively charged arginine) or the hydrophobicity (e.g., hydrophobic isoleucine to hydrophilic threonine). All increase the time course of inactivation, resulting in a net current increase. Taken together the amino acid substitutions at these three positions are likely to have a similar hypermorphic effect across all channels. Similarly, mutations to the S4 VSDs result in similar functional consequences across the channels (Figure 2C). The functionally analyzed hypomorphs are due to the loss of the positively charged arginine residue, which delays the channel’s opening and reduces the net current. These charged residues are highly conserved and sensitive to change that even the arginine substitution to another positively charged amino acid (histidine) has a deleterious effect in Cav1.1 and Cav1.3. This is possibly because histidine has a shorter R-chain than arginine. Interestingly, in Cav1.4 the addition of a positive amino acid in this already charged region abolishes the channel’s currents (p.Gly1007Arg). These accumulative consequences demonstrate that the smallest charge difference effects the fine-tuning of the VSDs and have a similar hypomorphic effect for all Cav1 channels.

## Discussion

The different chromosomal location of the four Cav1 genes suggests that there are more similarities in the type of mutations in autosomal Cav1.1–1.3 genes than there are when comparing all Cav1 genes; Cav1.1–1.3 are predominantly associated with missense mutations (81–85%), whereas missense mutations in Cav1.4 account for 35% of all mutations (Figure 1). Missense mutations in the autosomal Cav1 channels mostly result in hyperactive channels and manifest dominant Mendelian diseases. This implies that in dominant disorders the main disease mechanism is likely to be via GoF rather than LoF/haploinsufficiency. As Cav1 channels are heteromers, to decrease the function the mutant α-subunit protein must aggregate with the wildtype protein or preferentially bind with the interacting protein subunits to reduce their binding to the wildtype α-subunit protein.

Conversely, for Cav1.4, hypomorphic and null alleles are associated with recessive X-linked disease. This explains the high incidence of LoF mutations in Cav1.4 where there is no compensating wildtype allele in hemizygous males, underlining the observation that these are ‘hypomorphic phenotypes’, which suggests that LoF is likely the predominant disease mechanism in Cav1.4 channelopathies. Nevertheless, a small number of *CACNA1F* mutations result in increased overall calcium currents by increasing the current density or by causing larger currents at more hyperpolarized potentials (Hemara-Wahanui et al., 2005; Hoda et al., 2005; Peloquin et al., 2007). For example, the p.Il756Thr mutation is electrophysiologically a GoF mutation as it shifts the voltage dependence of activation to more negative potentials and reduced the inactivation, however, in the retina it causes a LoF with respect to the retinal function by damaging photoreceptors signaling to bipolar cells (second-order neurons) (Hemara-Wahanui et al., 2005).

The large number (85) of mutations in the C-terminal tail indicate that mutations in this important regulatory region disturb proper channel inactivation, and so, tissue function (Figure 2A). The morphological changes required for the inactivation of the channels are controlled by the voltage-dependent inactivation (VDI) and CDI. Transient calcium influx is controlled by CDI via the proximal C-terminal regulatory domains (PCRD) that require the coupling of the IQ and pre-IQ calmodulin-binding domains to an EF hand domain motif when calmodulin (CaM) is bound by four calcium ions (Hulme et al., 2006; Singh et al., 2006). As neurotransmitting photoreceptors require a sustained calcium influx the CDI in Cav1.4 is inhibited by distal C-terminal regulatory domain (DCRD), although, the binding site and precise mechanism is currently unknown. This negative inhibitory CDI (ICDI) feedback mechanism ensures that Cav1.4 is non-inactivating. The absence of CDI is unique to Cav1.4 and changes the voltage dependencies (electrophysiologically recorded as a leftward shift of the IV curve which changes the steepness, relative to Cav1.1–1.3) (Singh et al., 2006). The domain differences of this region between the channels suggest that mutations in Cav1.1–1.3 PCRD are likely to dysregulate the inactivation properties, which prevents transient influxes. In Cav1.4 the functional consequence is dependent on whether the mutation is in the PCRD or DCRD tail regions; mutations in the CDI may be tolerated to a degree as it is usually inhibited by ICDI, whereas mutations in the ICDI are likely to have a hypomorphic effect by preventing the inhibition of CDI and consequently allowing the channel to close prematurely.

From this analysis, we predict that there are similar functional consequences across Cav1 channels. For example, mutations in specific residues (Cav1.4: p.Gly369, p.Phe753, p.Ile756 in S6, Figure 2B) will have similar hypermorphic effects, and mutations in the VSDs will have similar hypomorphic consequences (Figure 2C). The extent of these effects is mutation specific.

There are some conflicting studies of *CACNA1F* mutations. One study reported no electrophysiological changes in a missense (p.Gly369Asp) and nonsense (p.Trp1459^∗^) mutation (McRory et al., 2004), whereas a second study reported both to be deleterious (Hoda et al., 2005). Our analyses suggest that both are likely to be pathogenic. The p.Gly369Asp mutation is consistent with the equivalent glycine residue being pathogenic in Cav1.2 and Cav1.3 (Figure 2B) and is predicted to be damaging by PolyPhen-2 (Supplementary Table 1). No protein expression was found for p.Trp1459^∗^ channels (Hoda et al., 2005), probably due to NMD. Functional investigations into these mutations and variants within close proximity will enhance these predictions and validate their pathogenicity.

The diverse functional consequences of Cav1 mutations suggests that certain therapies will be more pertinent to different channel dysfunctions. Over 60% of Cav1 pathogenic mutations are missense mutations, and 40% of these are associated with hypomorphic or null alleles. The differences between autosomal and X-linked channels suggest that therapeutic approaches for Cav1.4 X-linked iCSNB will differ from Cav1.1–1.3 channelopathies since the predominant mechanism for X-linked iCSNB is LoF. Treating GoF variants will require a different approach such as the use of inhibitors that act locally and specifically to inhibit or reduce the excess function. Although a reduced function as a consequence of protein dysregulation could potentially be corrected, a complete LoF can be embryonically lethal for all Cav1 channels as reported in Cav1.2 α_1_ knockout mice which died in utero (Zamponi et al., 2015).

For X-linked iCSNB caused through null alleles, gene replacement therapy would compensate for the lack of channel expression by providing a functional product sufficient to restore neurotransmission to normal levels. However, when Cav1.4 channels with reduced function are present then augmentation therapy may be more appropriate. Specific modulation drugs target different protein defects. Hypomorphs causing trafficking abnormalities and protein instability may be amenable to modulation drugs that increase or decrease protein expression, stabilize the protein, and open or close the channel gate. Drug “correctors” can stabilize protein folding and trafficking to the cell surface [e.g., elexacaftor or tezacaftor used to modulate the cystic fibrosis transmembrane conductance regulator protein (CFTR; Hoy, 2019)], or drug ‘potentiators’ that can bind proteins that are correctly localised but inactive and help them open to restore ion influx [e.g., ivacaftor used to activate mutant CFTR (Van Goor et al., 2009)]. As for cystic fibrosis, a combination therapy of both correctors and potentiators may be effective to facilitate protein stability, trafficking, and channel activation.

## Conclusion

Although Cav1 channels function in a similar way, genetic mutations have distinct consequences depending on whether the inheritance pattern is autosomal or X-linked. Functional *in vitro* studies have shown that a change in normal function that either increases or decreases conductance is pathogenic. Understanding the different mechanistic effect of Cav1 mutations reveals that effective therapeutic approaches for X-linked Cav1.4 diseases will be different from autosomal Cav1.1–1.3 channelopathies.

The Cav1.4 α_1_ gene has been known for over 20 years and a number of studies have contributed to our understanding of how this channel functions. Since its discovery, nearly 300 pathogenic mutations (92 missense) in *CACNA1F* have been cataloged, with functionally analyzed mutations affecting the channel’s ability to conduct calcium ions. Our analyses clarify the genetic mechanisms driving Cav1.4 channelopathies and differentiate the types of mutation and effect they have on channel function. This has highlighted that some mutated residues or domains are likely to have similar hypomorphic or hypermorphic effects across all Cav1 channels.

Our analyses have shown that LoF is the predominant mechanism causing Cav1.4 channelopathies through missense and truncating variations. However, as the number of variants that have been functionally analyzed in Cav1.4 is small, further studies are warranted to better understand the potentially treatable mutation types, as well as facilitating the prediction of variants of unknown significance identified during molecular diagnosis.

## Data Availability Statement

The original contributions presented in the study are included in the article/Supplementary Material, further inquiries can be directed to the corresponding author/s.

## Author Contributions

All authors listed have made a substantial, direct and intellectual contribution to the work, and approved it for publication.

## Conflict of Interest

The authors declare that the research was conducted in the absence of any commercial or financial relationships that could be construed as a potential conflict of interest. The handling editor declared a past co-authorship with one of the authors GB.

## Footnotes

1 https://www.ncbi.nlm.nih.gov/Class/Structure/aa/aa_explorer.cgi
