# Supplementary material for: A Review of Genetic and Physiological Disease Mechanisms Associated With Cav1 Channels: Implications for Incomplete Congenital Stationary Night Blindness Treatment
Source: Front Genet. 2021 Jan 28;12:637780. doi: 10.3389/fgene.2021.637780 (PMC7876387; doi:10.3389/fgene.2021.637780)
Supplement: Supplementary file 1 [file Data_Sheet_1.PDF]

Supplementary Materials

Supplementary Tables

Diagram representing the trimeric Cav1.4 channel is shown in Figure S1.

Each channel's mutations are plotted on a cartoon diagram for Cav1.1 (Figure S2, A), Cav1.2 (Figure S2, B), Cav1.3 (Figure S2, C), and Cav1.4 (Figure S2, D). The detailed analysis of each mutation is presented (Table S1).

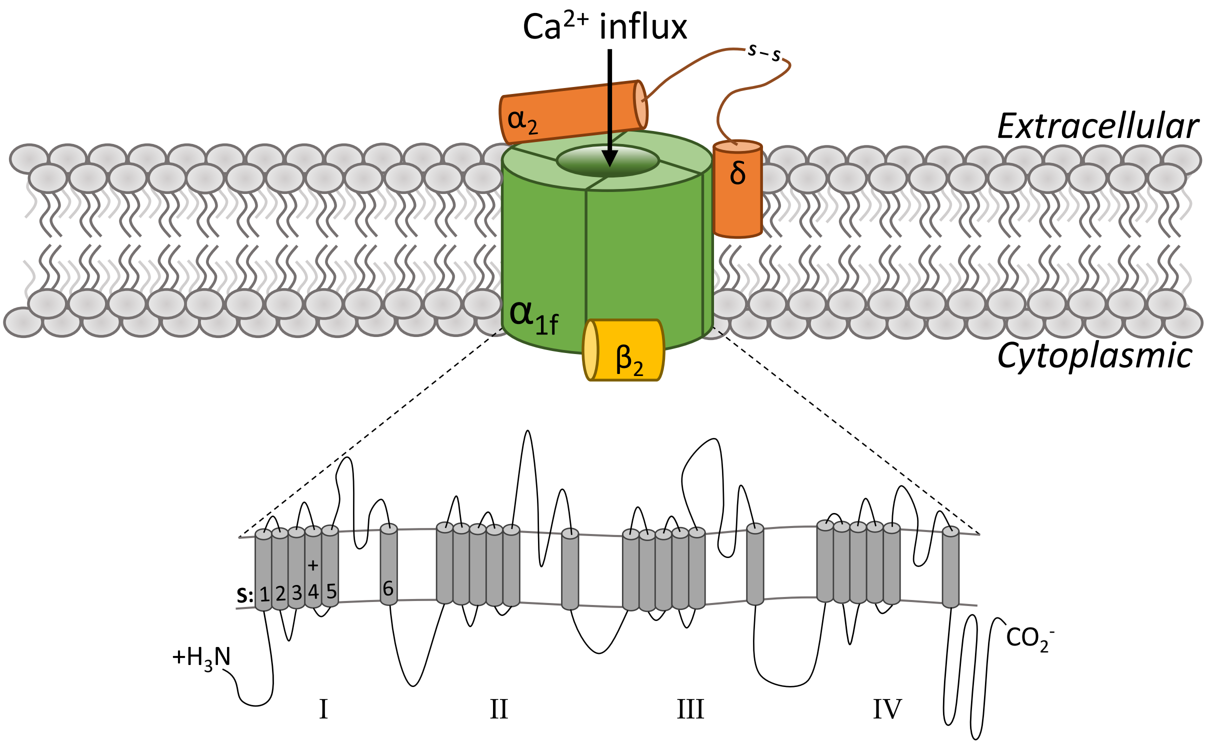

Figure S1. Diagram of the Cav1.4 transmembrane proteins. The schematic shows the trimeric Cav1.4 channel (top) and highlights the α_{1f} subunit structure (bottom). The four repeats (I-IV) and the α-helix segments (S: 1-6) of the α_{1f} subunit are labelled. The arrow shows the direction of calcium influx.

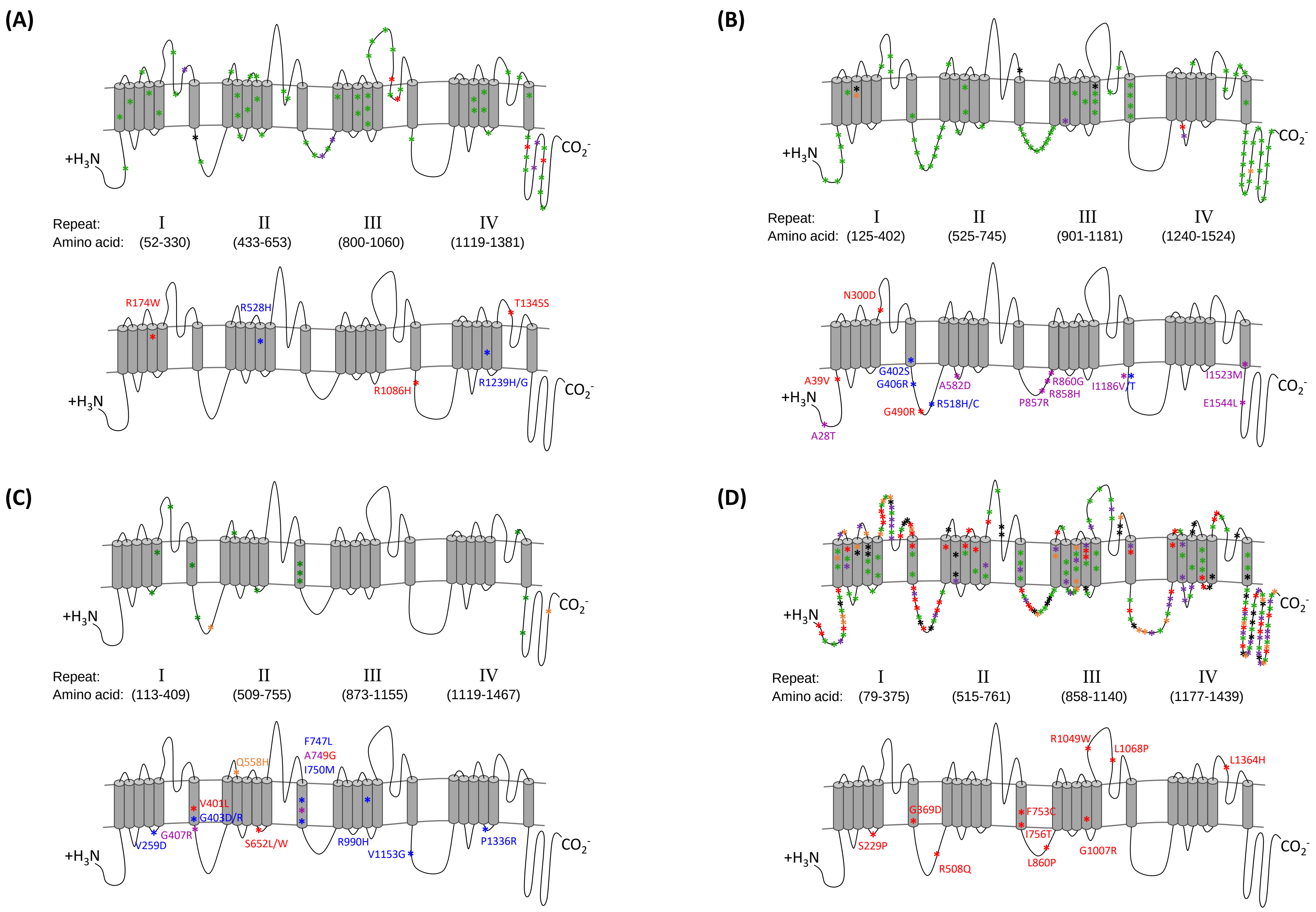
(A)
+H3N
CO2-
Repeat: I II III IV
Amino acid: (52-330) (433-653) (800-1060) (1119-1381)
R174W
R528H
T1345S
R1086H
R1239H/G
+H3N
CO2-
(B)
+H3N
CO2-
Repeat: I II III IV
Amino acid: (125-402) (525-745) (901-1181) (1240-1524)
N300D
A39V
G402S
G406R
A582D
R860G
R858H
I1186V/T
I1523M
R518H/C
G490R
P857R
E1544L
A28T
+H3N
CO2-
(C)
+H3N
CO2-
Repeat: I II III IV
Amino acid: (113-409) (509-755) (873-1155) (1119-1467)
F747L
A749G
I750M
Q558H
V401L
G403D/R
G407R
V259D
S652L/W
R990H
V1153G
P1336R
+H3N
CO2-
(D)
+H3N
CO2-
Repeat: I II III IV
Amino acid: (79-375) (515-761) (858-1140) (1177-1439)
R1049W
L1068P
L1364H
G369D
F753C
I756T
S229P
R508Q
L860P
G1007R
+H3N
CO2-

Figure S2. Cav1α gene mutations plotted on a channel cartoons. Stars represent a mutated residue and may include multiple amino acid substitution. Repeats (I-IV) and amino acid residues are labelled per channel. Top panels correspond to mutations reported on HGMD (missense; green, nonsense; red, splicing; black, insertions/ duplication; yellow) and bottom panels show missense mutations that have been functionally analysed. **(A)** The 66 *CACNA1S* mutations reported on HGMD (top) and 6 functionally analysed mutations (bottom). (Blue) hypokalaemia periodic paralysis mutations (3 hypomorphs), (red) malignant hyperthermia mutations (2 hypomorphs and 1 hypermorph). **(B)** The 85 *CACNA1C* mutations reported on HGMD (top) and 17 functionally analysed mutations (bottom). (Blue) Timothy syndrome mutations (2 hypomorphs and 3 hypermorphs), (red) Brugada syndrome 3 mutations (3 hypomorphs), (purple) long QT syndrome 8 mutations (9 hypermorphs). **(C)** The 16 *CACNA1D* mutations reported on HGMD (top) and 14 functionally analysed mutations (bottom). (Blue) aldosterone-producing adenomas mutations (1 hypomorph and 7 hypermorphs), (red) autism spectrum disorder mutations (4 hypermorphs), (orange) hearing impairment with intellectual disability mutations (1 hypomorph), (purple) primary aldosteronism, seizures, and neurologic abnormalities mutations (2 hypermorphs). **(C)** The 261 *CACNA1F* mutations reported on HGMD (top) and 10 functionally analysed mutations (bottom). (Red) iCSNB mutations (7 hypomorphs and 3 hypermorphs).

Table S1: Cav1 functionally analysed mutation analysis. iCSNB: congenital stationary night blindness, LQT8: long QT syndrome 8, BRGDA3: Brugada syndrome 3, TS: Timothy syndrome, MH: malignant hyperthermia, HypoPP: hypokalaemia periodic paralysis, APAs: aldosterone-producing adenomas, ASD: autism spectrum disorder, and PASNA: primary aldosteronism, seizures, and neurologic abnormalities. GoF: gain-of-function and LoF: loss-of-function. VDI: voltage-dependent inactivation. Vmax is the maximum ion currents recorded. Residue nomenclature may differ from seminal article based on transcript used.

Mutation	Phenotype	Mutation class	Conserved orthologue	Conserved paralogue	PolyPhen-2	Electrophysiological effect	Protein expression effect	Mutation effect	DOI
**Cav1.1 (*CACNA1S*)**									
**Arg174Trp**	MH	null	high	high	1	No currents.	-	LoF: no currents.	10.1016/j.bpj.2013.03.035
**Arg528His**	HypoPP	hypomorph	moderate	high	1	Reduced Vmax with slower activation.	-	LoF: reduced currents.	10.1523/JNEUROSCI.18-24-10320.1998
**Arg1086His**	MH	hypomorph	high	high	1	Reduced Vmax.	-	LoF: reduced currents.	10.1152/ajpcell.00173.2004
**Arg1239His**	HypoPP	hypomorph	high	high	0.98	Reduced Vmax with slower activation.	-	LoF: reduced currents.	10.1111/j.1469-7793.1999.00321.x
**Arg1239Gly**	HypoPP	hypomorph	-	-	0.98	Reduced Vmax with slower activation.	-	LoF: reduced currents.	10.1111/j.1469-7793.1999.00321.x
**Thr1345Ser**	MH	hypermorph	low	low	0.63	Increased activation.	-	GoF: increased current window.	10.1152/ajpcell.00008.2010
**Cav1.2 (*CACNA1C*)**									
**Ala28Thr**	LQT8	hypermorph	moderate	moderate	0.3	Depolarised shift in VDI and Vmax increased.	-	GoF: increased currents.	10.1016/j.yjmcc.2015.01.002
**Ala39Val**	BRGDA3	hypomorph	high	low	0.99	Reduced Vmax.	Perinuclear region.	LoF: reduced due to reduced membrane expression.	10.1161/CIRCULATIONAHA.106.668392
**Asn300Asp**	BRGDA3	hypomorph	high	low	0.99	Reduced Vmax.	Reduced membrane expression.	LoF: reduced due to reduced membrane expression.	10.1007/s00395-014-0446-5
**Gly402Ser**	TS	hypermorph	high	high	1	Slow inactivation.	-	GoF: increased current window.	10.1073/pnas.0502506102
**Gly406Arg**	TS	hypermorph	high	high	1	Slow inactivation and slight hyperpolarisation shift.	-	GoF: increased current window.	10.1073/pnas.0502506102
**Gly490Arg**	BRGDA3	hypomorph	moderate	moderate	0.76	Reduced Vmax.	Normal.	LoF: reduced currents.	10.1161/CIRCULATIONAHA.106.668392
**Arg518Cys**	TS	hypomorph	high	high	1	Reduced Vmax and slow inactivation.	Reduced membrane expression.	LoF: reduced due to reduced membrane expression.	10.1161/CIRCEP.115.002745

**Arg518His**	TS	hypomorph	-	-	1	Reduced Vmax and slow inactivation.	Reduced membrane expression.	LoF: reduced due to reduced membrane expression.	10.1161/CIRCEP.115.002745
**Ala582Asp**	LQT8	hypermorph	high	moderate	1	Depolarised inactivation shift.	-	GoF: increased current window.	10.1093/europace/euu063et al., 2014
**Pro857Arg**	LQT8	hypermorph	high	moderate	1	Increased Vmax.	Increased membrane expression.	GoF: increased currents due to more membrane expression.	10.1161/CIRCGENETICS.113.000138
**Arg858His**	LQT8	hypermorph	high	moderate	1	Increased Vmax and depolarised inactivation shift.	-	GoF: increased current window and larger currents.	10.1093/europace/euu063
**Arg860Gly**	LQT8	hypermorph	high	moderate	1	Depolarised shift in VDI.	-	GoF: increased current window.	10.1016/j.yjmcc.2015.01.002
**Ile1186Thr**	LQT8	hypermorph	high	high	0.99	Hyperpolarised shift and Vmax increased.	-	GoF: increased current window and larger currents.	10.1016/j.yjmcc.2015.01.002
**Ile1186Thr**	TS	hypermorph	-	-	-	Reduced Vmax and hyperpolarised shift in activation.	-	GoF: left shit increasing window current although reduced Vmax.	10.1016/j.hrthm.2014.09.051
**Ile1186Val**	LQT8	hypermorph	-	-	0.97	Vmax increased.	-	GoF: increased currents.	10.1016/j.yjmcc.2015.01.002
**Ile1523Met**	LQT8	hypermorph	moderate	high	0.72	Hyperpolarised shift in Vmax.	-	GoF: increased current window.	10.1016/j.yjmcc.2015.01.002
**Glu1544Lys**	LQT8	hypermorph	high	high	1	Hyperpolarised shift in Vmax and slowed VDI.	-	GoF: increased current window.	10.1016/j.yjmcc.2015.01.002
**Cav1.3 (*CACNA1D*)**									
**Val259Asp**	APAs	hypermorph	high	high	1	Hyperpolarised shift in Vmax and slow inactivation.	-	GoF: increased current window.	10.1038/ng.2716
**Val401Leu**	ASD and epilepsy	hypermorph	high	high	0.91	Increased Vmax, hyperpolarised shift and slow inactivation.		GoF: increased currents and current window.	10.1093/hmg/ddx175
**Gly403Asp**	APAs	hypermorph	high	high	1	Hyperpolarised shift in Vmax and slow inactivation.	-	GoF: increased current window.	10.1038/ng.2695
**Gly403Arg**	APAs	hypermorph	-	-	1	Hyperpolarised shift in Vmax and slow inactivation.	-	GoF: increased current window.	10.1038/ng.2695
**Gly407Arg**	PASNA	hypermorph	high	high	0.98	Hyperpolarised shift in Vmax and slow inactivation.	-	GoF: increased current window.	10.1016/j.biopsych.2014.11.020
**Gln558His**	Hearing & intellectual impairment	hypomorph	high	high	0.99	Decreased Vmax.	Reduced membrane expression.	LoF: reduced due to reduced membrane expression.	10.1074/jbc.RA118.003681
**Ser652Leu**	ASD	hypermorph	high	high	0.99	Hyperpolarised shift in activation and inactivation but reduced currents.	-	GoF: increased current window.	10.1186/s13229-019-0310-4
**Ser652Trp**	ASD	hypermorph	high	high	0.99	Hyperpolarised shift in activation and inactivation.	-	GoF: increased current window.	10.1186/s13229-019-0310-4

**Phe747Leu**	APAs	hypermorph	high	high	1	Hyperpolarised shift in activation.	-	GoF: increased current window.	10.1080/19336950.2018.1546518
**Ala749Gly**	PASNA	hypermorph	high	high	1	Hyperpolarised shift in Vmax.	-	GoF: increased current window.	10.1016/j.biopsych.2014.11.020
**Ala749Gly (Ala760Gly)**	ASD	hypermorph	-	-	-	Hyperpolarised shift, decreased CDI and slow inactivation.	-	GoF: increased current window.	10.1038/srep27235
**Ile750Met**	APAs	hypermorph	high	high	1	Hyperpolarised shift in Vmax and slow inactivation.	-	GoF: increased current window.	10.1038/ng.2695 / 10.1038/ng.2716
**Arg990His**	APAs	hypomorph	high	high	1	Depolarised shift in activation and reduced Vmax.	-	LoF: decreased currents.	10.1016/j.bpj.2017.08.010
**Val1153Gly**	APAs	hypermorph	moderate	high	0.99	Hyperpolarised shift in Vmax and slow inactivation.	-	GoF: increased current window.	10.1161/HYPERTENSIONAHA.117.09057
**Pro1336Arg**	APAs	hypermorph	moderate	low	1	Slow inactivation.	-	GoF: increased current window.	10.1038/ng.2716
**Cav1.4 (*CACNA1F*)**									
**Ser229Pro**	iCSNB	null	high	high	1	No currents.	Normal.	LoF: no currents.	10.1523/JNEUROSCI.3054-04.2005
**Gly369Asp**	iCSNB	hypermorph	high	high	1	Hyperpolarised shift in Vmax and slowed inactivation.	Normal.	GoF: increased window current.	10.1523/JNEUROSCI.3054-04.2005
**Gly369Asp**	iCSNB	-	-	-	-	Depolarised shift in Vmax.	-	No change.	10.1523/JNEUROSCI.4846-03.2004
**Arg508Gln**	iCSNB	hypomorph	high	moderate	0.02	Normal currents.	Reduced protein expression.	LoF: less protein expressed.	10.1111/j.1471-4159.2006.03678.x
**Phe753Cys**	iCSNB	hypermorph	high	high	0.88	Hyperpolarised shift in Vmax and slower inactivation.	-	GoF: increased window current.	10.1016/j.neuroscience.2007.09.021
**Ile756Thr**	iCSNB	hypermorph	low	high	1	Hyperpolarised shift in Vmax and slower inactivation.	-	GoF: increased window current.	10.1073/pnas.0501907102
**Leu860Pro**	iCSNB	hypomorph	moderate	low	0.92	Reduced Vmax.	Normal.	LoF: reduced currents.	10.1016/j.bbamem.2014.04.023
**Gly1007Arg**	iCSNB	null	high	high	1	No currents.	Normal.	LoF: no currents.	10.1016/j.neuroscience.2007.09.021
**Arg1049Trp**	iCSNB	null	moderate	high	0.99	No currents.	Normal.	LoF: no currents.	10.1016/j.neuroscience.2007.09.021
**Leu1068Pro**	iCSNB	null	high	high	0.97	No currents.	Normal.	LoF: no currents.	10.1523/JNEUROSCI.3054-04.2005

**Leu1364His**	iCSNB	hypomorph	high	high	0.87	Normal currents.	Reduced protein expression.	LoF: less protein expressed.	10.1111/j.1471-4159.2006.03678.x
**Trp1459* (1440)**	iCSNB	null	high	high	-	No currents.	No protein expression.	LoF: no currents.	10.1523/JNEUROSCI.3054-04.2005
**Lys1591***	iCSNB	hypomorph	low	moderate	-	Hyperpolarised shift in Vmax.	-	LoF: reduced currents.	10.1038/nn1751
**Arg1827***	iCSNB	conflicting hyper- and hypo-morph	low	low	-	Hyperpolarised shift in Vmax and reduced Vmax.	Normal.	GoF: left shift but slight LoF: reduced currents.	10.1016/j.bbamem.2014.04.023
